# Supplementary material for: Distributable, metabolic PET reporting of tuberculosis
Source: Nat Commun. 2024 Jun 27;15:5239. doi: 10.1038/s41467-024-48691-6 (PMC11211441; doi:10.1038/s41467-024-48691-6)
Supplement: Supplementary file 3 — Reporting Summary [file 41467_2024_48691_MOESM3_ESM.pdf]

nature portfolio

Corresponding author(s): Davis, Barry III

Last updated by author(s): Apr 17, 2024

Reporting Summary

Nature Portfolio wishes to improve the reproducibility of the work that we publish. This form provides structure for consistency and transparency in reporting. For further information on Nature Portfolio policies, see our Editorial Policies and the Editorial Policy Checklist.

Statistics

For all statistical analyses, confirm that the following items are present in the figure legend, table legend, main text, or Methods section.

n/a	Confirmed	
[ ]	[x]	The exact sample size (*n*) for each experimental group/condition, given as a discrete number and unit of measurement
[ ]	[x]	A statement on whether measurements were taken from distinct samples or whether the same sample was measured repeatedly
[ ]	[x]	The statistical test(s) used AND whether they are one- or two-sided<br>*Only common tests should be described solely by name; describe more complex techniques in the Methods section.*
[x]	[ ]	A description of all covariates tested
[x]	[ ]	A description of any assumptions or corrections, such as tests of normality and adjustment for multiple comparisons
[ ]	[x]	A full description of the statistical parameters including central tendency (e.g. means) or other basic estimates (e.g. regression coefficient) AND variation (e.g. standard deviation) or associated estimates of uncertainty (e.g. confidence intervals)
[ ]	[x]	For null hypothesis testing, the test statistic (e.g. *F*, *t*, *r*) with confidence intervals, effect sizes, degrees of freedom and *P* value noted<br>*Give P values as exact values whenever suitable.*
[x]	[ ]	For Bayesian analysis, information on the choice of priors and Markov chain Monte Carlo settings
[x]	[ ]	For hierarchical and complex designs, identification of the appropriate level for tests and full reporting of outcomes
[x]	[ ]	Estimates of effect sizes (e.g. Cohen's *d*, Pearson's *r*), indicating how they were calculated

Our web collection on statistics for biologists contains articles on many of the points above.

Software and code

Policy information about availability of computer code

Data collection	Scan data were collected with a Mediso LFER150 PET/CT, and tissue distribution data were collected a Siemens Pet/CT scanner and with a Wallac Wizard Gamma Counter (Perkin Elmer, 946 Waltham, MA). LC and general additional chromatography and flow methods using Lab solutions CS (Shimadzu), Smart Control Software (Version 6.20, BMG Labtech), Smart Control MARS (Version 4.2, BMG Labtech), UNICORN (version 5.31), Agilent Chemstation.
Data analysis	Enzyme kinetics using GraphPad Prism 8 (Graphpad software Inc.). Scan data using MIM Maestro (MIM Software Inc, Cleveland, OH USA), OLINDA 2.1 (Vanderbilt University, Nashville TN). MS data using MassLynx software (v. 4.1 from Waters). NMR data using Topspin 4 (Bruker biospin GmbH) and MestReNova 14 (Mestrelab Research S.L). Additional general use of Microsoft Excel (Microsoft).

For manuscripts utilizing custom algorithms or software that are central to the research but not yet described in published literature, software must be made available to editors and reviewers. We strongly encourage code deposition in a community repository (e.g. GitHub). See the Nature Portfolio guidelines for submitting code & software for further information.

Data

Policy information about availability of data

All manuscripts must include a data availability statement. This statement should provide the following information, where applicable:

- Accession codes, unique identifiers, or web links for publicly available datasets
- A description of any restrictions on data availability
- For clinical datasets or third party data, please ensure that the statement adheres to our policy

Raw NMR spectra, small molecules and protein LC-MS raw data, sequence files and radio-HPLC raw data has been deposited in the Zenodo repository (10.5281/zenodo.10624807). PET-CT data related to infected and naïve marmosets has been deposited into Accessclinical data@NIAID with the link: https://accessclinicaldata.niaid.nih.gov/study-viewer/clinical_trials/MARM-TB-FDT.

Research involving human participants, their data, or biological material

Policy information about studies with human participants or human data. See also policy information about sex, gender (identity/presentation), and sexual orientation and race, ethnicity and racism.

Reporting on sex and gender	N/A
Reporting on race, ethnicity, or other socially relevant groupings	N/A
Population characteristics	N/A
Recruitment	N/A
Ethics oversight	N/A

Note that full information on the approval of the study protocol must also be provided in the manuscript.

Field-specific reporting

Please select the one below that is the best fit for your research. If you are not sure, read the appropriate sections before making your selection.

- [x] Life sciences
- [ ] Behavioural & social sciences
- [ ] Ecological, evolutionary & environmental sciences

For a reference copy of the document with all sections, see nature.com/documents/nr-reporting-summary-flat.pdf

Life sciences study design

All studies must disclose on these points even when the disclosure is negative.

Sample size	No sample size calculations were made as labeling experiments were demonstrations of performance and not of statistical difference between groups of animals. In most cases, an animal served as its own control as it was imaged with both of the probes to be compared.
Data exclusions	No data were excluded unless an animal expired before data was collected on both probes or on both blocked and unblocked conditions.
Replication	For enzyme analyses typical triplicates unless otherwise stated; all attempts were successful. Imaging experiments were repeated on separate days with newly synthesized probes to compare consistency of labeling. At least 2 biological replicates were collected for each animal experiment; all attempts at animal experiment replication were successful with the exception that if an animal was to sick to undergo an additional set of PET/CT scans and had to be humanely euthanized.
Randomization	There were no groups of animals that required randomization. The order of probe application was randomized as to which received i.e. the blocking agent first (for example by Animal alphabetical ID), but then 2 days later the application of the block agent or probe type was reversed so that animals were imaged under both conditions.
Blinding	Readers of images were blind to treatment assignment during analysis and compilation of the quantitative data. Group assignment was revealed only after data extraction was complete.

Reporting for specific materials, systems and methods

We require information from authors about some types of materials, experimental systems and methods used in many studies. Here, indicate whether each material, system or method listed is relevant to your study. If you are not sure if a list item applies to your research, read the appropriate section before selecting a response.

Materials & experimental systems

n/a	Involved in the study	
☐	☒	Antibodies
☒	☐	Eukaryotic cell lines
☒	☐	Palaeontology and archaeology
☐	☒	Animals and other organisms
☒	☐	Clinical data
☒	☐	Dual use research of concern
☒	☐	Plants

Methods

n/a	Involved in the study	
☒	☐	ChIP-seq
☒	☐	Flow cytometry
☒	☐	MRI-based neuroimaging

Antibodies

Antibodies used	OtsA and B detection: Primary : Mouse Anti-6x-His Antibody - Invitrogen # MA1-135<br>https://www.thermofisher.com/antibody/product/6x-His-Tag-Antibody-clone-4E3D10H2-E3-Monoclonal/MA1-135<br>Secondary : Goat Anti-Mouse IgG-AP Conjugate - Bio-Rad #1706520<br>https://www.bio-rad.com/en-us/sku/1706520-goat-anti-mouse-igg-ap-conjugate?ID=1706520<br>Hexokinase deception: Primary : RABBIT ANTI HEXOKINASE - Bio-Rad # 4959-9988<br>Secondary : SHEEP ANTI RABBIT IgG:DyLight®488 - Bio-Rad # STAR36D488GA
Validation	Anti-6x-His validated by ThermoFisher: "This Antibody was verified by Relative expression to ensure that the antibody binds to the antigen stated." [https://www.thermofisher.com/antibody/product/6x-His-Tag-Antibody-clone-4E3D10H2-E3-Monoclonal/MA1-135]<br>Antihexokinase validated by BioRad: "This product has been reported to work in the following applications. This information is derived from testing within our laboratories, peer-reviewed publications or personal communications from the originators."

Animals and other research organisms

Policy information about studies involving animals; ARRIVE guidelines recommended for reporting animal research, and Sex and Gender in Research

Laboratory animals	Mice: C57BL/6 adult female; Rabbits NZW adult females; common marmosets - outbred adults in bred NIH facilities of both sexes
Wild animals	No wild animals were used in this study.
Reporting on sex	No reporting of sex has been included as the number of animals was small and so sex differences were explored.
Field-collected samples	No field collected samples were used in this study.
Ethics oversight	This study was carried out in accordance with the recommendations in the Guide for the Care and Use of Laboratory Animals of the National Institutes of Health. Biodistribution studies with naive rhesus macaques were approved by the Institutional Animal Care and Use Committee (IACUC) of the NIH Clinical Centre (Bethesda, MD). The IACUC of the NIAID, NIH approved the experiments described herein with mice rabbits and marmosets under protocols LCID-3 -4 and LCID-9 respectively (Permit issued to NIH Intramural Research Program as A-4149-01). FDT studies in three cynomolgus macaque at University of Pittsburgh were approved by its IACUC and Division of Radiation Safety.

Note that full information on the approval of the study protocol must also be provided in the manuscript.

Plants

Seed stocks	N/A
Novel plant genotypes	N/A
Authentication	N/A
